# Recent advances in coronary in-stent restenosis: a comprehensive narrative review

**DOI:** 10.3389/fcvm.2026.2002465

**Published:** 2026-10-27

**Authors:** Mingliang Du, Hui Hui

**Affiliations:** Department of Coronary Heart Disease, Central Hospital of Dalian University of Technology (Dalian Municipal Central Hospital), Liaoning, China

**Keywords:** drug-coated balloon, drug-eluting stent, in-stent restenosis, intravascular imaging, neoatherosclerosis, percutaneous coronary intervention

## Abstract

Coronary in-stent restenosis (ISR) remains an important limitation of percutaneous coronary intervention despite the use of drug-eluting stents. ISR is a heterogeneous process driven by neointimal hyperplasia, neoatherosclerosis, and mechanical factors such as stent underexpansion or fracture. Intravascular imaging, particularly IVUS and OCT, enables mechanism-guided lesion preparation and device selection, while metabolomics, molecular imaging, and circulating biomarkers are improving understanding of its biological heterogeneity. Adequate lesion preparation is fundamental, and drug-coated balloons and repeat drug-eluting stent implantation remain the principal treatment strategies. Adjunctive techniques such as intravascular lithotripsy may help in severely calcified or resistant underexpanded lesions. Emerging evidence also implicates sphingolipid metabolism, mitochondrial dysfunction, immune-inflammatory signaling, and systemic metabolic modulation in ISR, though clinical relevance requires validation. This review summarizes recent advances in the epidemiology, pathophysiology, risk factors, diagnosis, treatment, and prevention of ISR, emphasizing the shift from anatomy-based management toward mechanism-guided, individualized therapy.

## 1 Introduction

Since Andreas Grüntzig performed the first percutaneous transluminal coronary angioplasty in 1977, PCI has undergone successive technological advances, progressing from plain balloon dilatation to bare-metal stents (BMS), first-generation drug-eluting stents (DES), and subsequently second-generation DES. Continued refinements in stent alloys, drug-coating formulations, and antiplatelet regimens have substantially reduced the need for clinically driven reintervention for in-stent restenosis. A pooled analysis of earlier randomized trials showed that, with the evolution of stent technology from BMS to early- and new-generation DES, the 1-year rate of ischemia-driven target lesion revascularization (TLR) decreased from 14.7% to 4.9% and 2.5%, respectively. Nevertheless, ischemia-driven TLR continued to accrue between 1 and 5 years, indicating that new-generation DES have markedly reduced, but not eliminated, the risk of repeat revascularization for late restenosis (1, 2).

At the same time, the high global volume of PCI means that the absolute burden of ISR remains substantial. In a meta-analysis of 12 studies involving 708,391 patients, 71,353 patients (10.3%) underwent PCI for ISR lesions (3). Compared with PCI for *de novo* lesions, PCI for ISR was associated with a higher risk of major adverse cardiac events (MACE; OR 1.31, 95% CI 1.18–1.46), as well as increased risks of target vessel revascularization (OR 1.42, 95% CI 1.29–1.55), myocardial infarction (OR 1.20), and stent thrombosis (OR 1.44), underscoring the distinct clinical challenges associated with ISR. In 2023, the Society for Cardiovascular Angiography and Interventions (SCAI) issued an expert consensus statement on the management of ISR and stent thrombosis (4), emphasizing the integration of intravascular imaging with the timing and underlying mechanism of ISR to guide subsequent treatment and thereby advancing ISR management toward a more precise, mechanism-based approach.

Several contemporary reviews and consensus documents have summarized the general diagnosis and treatment of ISR. The present review is positioned differently: it integrates landmark evidence that established DES and DCB therapy with evidence published mainly from 2020 through August 2026, including recent randomized DCB trials, intravascular-imaging registries, metabolomic studies, and emerging molecular targets. Its organizing framework is mechanism-guided management. Biological phenotypes (neointimal hyperplasia and neoatherosclerosis) and mechanical phenotypes (underexpansion, fracture, deformation, and geographic miss) are linked to lesion preparation and device selection. This framework is intended to move the discussion from a catalogue of technologies toward phenotype-specific, individualized treatment.

### 1.1 Literature search and evidence selection

For this narrative review, we performed a structured literature search in PubMed/MEDLINE from database inception through June 30, 2026. Search terms combined ‘coronary in-stent restenosis' or ‘ISR' with ‘pathophysiology,’ ‘neoatherosclerosis,’ ‘intravascular ultrasound,’ ‘optical coherence tomography,’ ‘drug-coated balloon,’ ‘drug-eluting stent,’ ‘recurrent restenosis,’ ‘brachytherapy,’ ‘excimer laser,’ ‘lithotripsy,’ ‘biomarker,’ and related terms. Reference lists of major trials, meta-analyses, consensus statements, and relevant reviews were also screened. We prioritized randomized trials and individual-patient-data meta-analyses for comparative treatment questions, prospective imaging studies and registries for mechanism and diagnosis, and primary translational studies for emerging biological pathways. Recent observational studies and small series were included only when they addressed technologies or clinical scenarios not represented in randomized evidence. Because this was a narrative rather than systematic review, study selection was guided by clinical relevance, methodological rigor, and contribution to the mechanism-guided framework; no formal risk-of-bias assessment or quantitative evidence synthesis was performed.

## 2 Epidemiology and clinical presentation

New-generation DES have substantially altered the epidemiology of ISR, but the pronounced time dependence of restenosis risk underscores the importance of long-term follow-up. As noted above, the 1-year TLR rate with new-generation DES is approximately 2.5%; however, this risk is not transient and continues to accumulate over time. Between 1 and 5 years, cumulative ischemia-driven TLR rates were 6.1%, 5.9%, and 4.4% for BMS, early-generation DES, and new-generation DES, respectively, such that the differences among the three groups narrowed appreciably (2). Thus, new-generation DES have not eliminated ISR but may instead delay its occurrence, highlighting the need for continued vigilance during long-term follow-up. In a retrospective cohort of 107 patients who underwent clinically indicated angiographic follow-up within 12 months after STEMI, Munteanu et al. (5) detected ISR of any degree in 87% of patients, with significant ISR (luminal stenosis >70%) present in 52%. Importantly, only patients with a clinical indication for repeat angiography were included; therefore, the cohort was subject to substantial selection bias, and these proportions cannot be extrapolated to the overall incidence of ISR in the broader STEMI population. Within this cohort, advanced age, previous cardiovascular events, diabetes, chronic kidney disease, and prior stroke were all associated with a significantly higher risk of ISR, while mortality among patients with severe ISR was 6%. When late ISR is complicated by rupture or erosion of neoatherosclerotic plaque, exposure of a prothrombotic substrate may precipitate thrombosis and manifest as an acute coronary syndrome. The clinical presentation ultimately depends on the underlying mechanism, the severity of stenosis, and the presence of thrombus (2, 4).

Recurrent ISR (R-ISR) represents an even greater clinical challenge. In a systematic review and meta-analysis of 23 studies, Chen et al. (6) reported a pooled incidence of R-ISR of 21.7% (95% CI, 17.1%–27.2%), with higher rates observed in European populations, lesions not treated with DES, and subgroups with a follow-up duration of ≤6 months. Significant risk factors included renal disease (OR 3.90; 95% CI, 1.87–8.11), non-focal restenosis (OR 3.29; 95% CI, 2.41–4.49), lesion length (OR 1.15; 95% CI, 1.06–1.24), a heterogeneous tissue pattern (OR 3.36; 95% CI, 1.69–6.67), stent fracture (OR 2.21; 95% CI, 1.29–3.79), and a higher percentage diameter stenosis (OR 1.05; 95% CI, 1.03–1.06). Collectively, these findings highlight the substantial propensity of ISR to recur and support careful identification of risk factors and mechanism-guided treatment at the time of reintervention.

## 3 Pathogenesis

### 3.1 Mechanical injury and endothelial dysfunction

The pathophysiology of ISR begins with mechanical injury to the vessel wall during stent implantation. Penetration and compression of the endothelium by stent struts cause extensive endothelial denudation and expose the subendothelial matrix, thereby promoting platelet adhesion and aggregation, activating the extrinsic coagulation cascade, and triggering a local inflammatory response through multiple signaling pathways. The extent and rate of re-endothelialization directly influence subsequent neointimal hyperplasia. An intact endothelial layer continuously releases protective mediators, such as nitric oxide and prostacyclin, that inhibit smooth muscle cell proliferation and inflammatory cell infiltration, whereas delayed or incomplete re-endothelialization leaves the vessel wall exposed to a proproliferative and proinflammatory environment for a prolonged period (7).

In a recent review, Yi et al. (7) organized the regulatory network underlying neointimal hyperplasia around three core processes—endothelial injury, VSMC phenotypic switching, and inflammation—and systematically catalogued the molecular mediators involved at each level, including proproliferative factors such as LSD1 and FGF10, protective mediators such as CGRP and A20, and targets involved in endothelial repair such as VEGF. VEGF contributes to endothelial proliferation and repair, but its effects on VSMCs are context dependent. At present, it is therefore more appropriately regarded as a candidate regulatory target for endothelial repair rather than an established therapeutic strategy based on “precise activation” (7). Stent design, strut thickness, and polymer biocompatibility also influence vascular healing and late stent-related events (2).

The historical evidence base also supports this sequence of injury, inflammation, and repair. Pathological studies of human coronary stents demonstrated that acute thrombus deposition and inflammation are followed by smooth-muscle-cell proliferation and extracellular-matrix accumulation, with the magnitude of arterial injury correlating with neointimal growth. Landmark SIRIUS and TAXUS-IV trials subsequently showed that local delivery of sirolimus or paclitaxel markedly reduced restenosis compared with bare-metal stents, clinically validating suppression of neointimal proliferation as an antirestenotic strategy (8, 9).

### 3.2 Phenotypic switching, proliferation and migration of vascular smooth muscle cells

Phenotypic switching of vascular smooth muscle cells (VSMCs) from a quiescent contractile phenotype to a highly active synthetic phenotype is a central cellular event in neointimal hyperplasia. Within the inflammatory microenvironment induced by stent-related vascular injury, VSMCs are driven by the concerted action of proproliferative signals, such as platelet-derived growth factor (PDGF), fibroblast growth factor (FGF), and transforming growth factor-*β* (TGF-*β*), to undergo extensive proliferation and migrate toward the intima while secreting extracellular matrix (ECM) proteins, ultimately resulting in progressive luminal narrowing.

Recent mechanistic studies have identified several novel pathways involved in the regulation of VSMC proliferation. An et al. (10) showed that nitrite increases mitofusin-1 (Mfn1) protein levels by inhibiting its proteasomal degradation, thereby arresting cell-cycle progression in rat aortic smooth muscle cells. Loss of Mfn1 enhanced proliferation, suppressed the expression of contractile genes, and downregulated antioxidant enzymes, including catalase and glutathione peroxidase, whereas restoration of antioxidant capacity substantially reversed these changes. In smooth muscle-specific Mfn1 knockout mice subjected to carotid artery ligation injury, neointimal hyperplasia was significantly greater than that in wild-type mice, and nitrite attenuated neointimal formation only in wild-type animals. These findings implicate mitochondrial dynamics—the balance between mitochondrial fusion and fission—as a novel regulatory target for controlling the VSMC phenotype. Similarly, Johnson and Trebak (11) documented a close association between altered ion channel activity and VSMC phenotypic switching and proliferation, providing a rationale for the development of antirestenotic therapies targeting ion channels.

### 3.3 Advances in inflammatory and immune mechanisms

Chronic local inflammation is a crucial link between mechanical injury and neointimal hyperplasia. The inflammatory cascade following stent implantation involves neutrophils, monocytes/macrophages, and lymphocytes, while the coordinated release of proinflammatory cytokines, including IL-1β, IL-6, and TNF-α, together with chemokines, creates a vicious cycle that amplifies proliferative signaling in VSMCs (7). In patients with diabetes, upregulation of IL-6 and its signaling pathway, together with increased expression of adhesion molecules such as VCAM-1, ICAM-1, and E-selectin, is considered an important mechanism through which hyperglycemia promotes the proliferation of intimal VSMCs (2).

The immunoregulatory role of B lymphocytes in ISR has more recently attracted considerable attention. In a clinical study of 812 patients undergoing PCI, including 237 patients with ISR and 575 without ISR, Yang et al. (12) found that both the percentage and absolute count of peripheral blood B lymphocytes were significantly higher in the ISR group (*P* = 0.011 and *P* = 0.010, respectively). On multivariable analysis, B-cell percentage (OR 1.033; 95% CI, 1.002–1.065), diabetes (OR 1.556), and non-high-density lipoprotein cholesterol (OR 1.210) were identified as independent risk factors for ISR. Subgroup analysis further indicated that a high B-cell percentage (≥12%) was associated with a greater risk in patients with diabetes, and that chronic hyperglycemia (HbA1c ≥ 6.6%) and B-cell levels showed a synergistic association with ISR. These observations suggest that B lymphocytes may participate in the immune mechanisms underlying diabetes-related ISR. However, the observational design of the study precludes causal inference and does not support B-cell-directed immunotherapy at present; both the underlying mechanisms and the potential therapeutic value of such an approach require further experimental investigation and prospective clinical validation.

Oxidative stress also occupies an important intermediate position along the inflammation–proliferation axis. Accumulation of reactive oxygen species (ROS) not only directly impairs endothelial mitochondrial function and promotes VSMC phenotypic switching but also establishes a positive feedback loop between inflammation and oxidative stress through activation of nuclear transcription factors such as NF-*κ*B. In a rat carotid artery balloon injury model, nitroxide radical-containing polymeric micelle nanoparticles selectively accumulated at the injured endothelium and penetrated the neointima and media, markedly reducing the intimal area and intima/media ratio. These effects were associated with suppression of ROS and oxidized low-density lipoprotein accumulation, a trend toward reduced LOX-1 expression, and decreases in E-selectin-positive endothelial cells and infiltrating M1 macrophages, with greater efficacy than that observed with the small-molecule antioxidant TEMPOL (13). However, translation of these findings from animal models to clinical application remains limited by challenges related to delivery efficiency and target-organ safety.

### 3.4 Lipids and novel molecular targets

Disordered lipid metabolism exerts an important regulatory influence on the development and progression of ISR, and recent multi-omics studies have provided new insights into this process. In a two-stage prospective cohort study of patients undergoing PCI for chronic total occlusion (CTO) lesions (discovery cohort, *n* = 161; independent validation cohort, *n* = 162; plasma sampled 48 h after the procedure; 9-month follow-up), Meng et al. (14) integrated plasma metabolomics, correlation network analysis, and machine learning to construct a network metabolite signature, NetMet-ISR, which predicted ISR with an area under the curve (AUC) of 0.935 and clearly outperformed individual metabolite markers. Pathway enrichment analysis revealed significant dysregulation of three sphingolipid-related pathways—ceramide metabolism, sphingomyelin metabolism, and deoxysphingolipid metabolism—centered on acid sphingomyelinase (sphingomyelin phosphodiesterase 1, SMPD1) as a key upstream enzyme. SMPD1 hydrolyzes lysosomal sphingomyelin to generate ceramide, which may impair endothelial repair and promote VSMC proliferation and migration through proapoptotic and proinflammatory signaling. In subsequent translational experiments, virtual screening identified the antihypertensive agent quinapril as an SMPD1 inhibitor, and quinapril significantly reduced neointimal area and percentage area stenosis in a porcine coronary stent model. These findings provide preliminary translational evidence that the SMPD1/ceramide axis may serve as both a biomarker and a potential therapeutic target. However, because the study population was restricted to patients with CTO lesions, the generalizability of these findings to the broader population undergoing PCI for coronary artery disease requires further validation.

The systematic application of metabolomics to biomarker discovery in ISR represents another important research direction. Using gas chromatography–mass spectrometry (GC-MS), Yan et al. (15) performed unbiased serum metabolomic profiling in 61 patients after PCI (18 with ISR and 43 without ISR) and identified seven differentially abundant metabolites: inosine, inositol, 3-cyanoalanine, glyceryl monostearate, and glutamine were upregulated, whereas biuret and 3-methylcatechol were downregulated. Pathway enrichment analysis implicated inositol phosphate metabolism, alanine–aspartate–glutamate metabolism, and the phosphatidylinositol signaling system. Receiver operating characteristic analysis showed that inosine had the best diagnostic performance among individual metabolites (AUC=0.807), whereas a combined biomarker panel achieved an AUC of 0.925. These metabolic signatures provide new insights into the metabolic basis of ISR and may support the development of serum metabolomics-based approaches for early risk stratification. However, this was a single-center retrospective study with a small sample size, and the findings require prospective validation in larger cohorts.

### 3.5 In-stent neoatherosclerosis

As the interval following DES implantation lengthens, an increasing proportion of ISR cases present as in-stent neoatherosclerosis, characterized by pathological changes resembling native atherosclerosis, including foam cell accumulation, lipid core formation, and fibrous cap thinning within the stent neointima. This process is an important substrate of late restenosis and differs fundamentally from early ISR, which is predominantly characterized by homogeneous neointimal hyperplasia. Optical coherence tomography (OCT) studies indicate that neoatherosclerosis is strongly time dependent and develops earlier in DES-ISR than in BMS-ISR. Pathological and intravascular imaging studies have further shown that neoatherosclerotic plaques within stents may undergo rupture or erosion and are associated with adverse events, including late stent failure, acute coronary syndrome, and stent thrombosis. From a therapeutic perspective, calcified neoatherosclerosis can reduce lesion compliance. In the presence of marked calcification or stent underexpansion, adequate lesion preparation should be performed under intravascular imaging guidance, with the choice among high-pressure non-compliant balloons, cutting or scoring balloons, and intravascular lithotripsy (IVL) guided by the underlying mechanism before subsequent treatment with a DCB or DES is selected (4). Autopsy evidence demonstrated that neoatherosclerosis occurs more frequently and substantially earlier after DES than after BMS implantation, establishing it as a distinct mechanism of late stent failure rather than simply prolonged neointimal growth (16).

## 4 Risk factors

### 4.1 Conventional patient-related risk factors

Diabetes is currently one of the best-established patient-level risk factors for ISR and promotes restenosis through several converging mechanisms. Sustained hyperglycemia leads to the accumulation of advanced glycation end products (AGEs), resulting in oxidative vascular injury and endothelial dysfunction; hyperinsulinemia associated with insulin resistance directly stimulates VSMC proliferation; diabetes-related platelet hyperreactivity aggravates thrombus formation after stent implantation; and the combined effects of B-lymphocyte activation and hyperglycemia may further amplify inflammatory signaling (12).

Chronic kidney disease (CKD) is another well-recognized independent risk factor for ISR. The persistent inflammatory state associated with CKD creates a milieu that predisposes to restenosis. Patients with CKD and ISR also more frequently present with ACS, and the risk of adverse events is highest among those with severe CKD (eGFR 15–29 mL/min/1.73 m^2^) or end-stage renal disease (2).

Dyslipidemia, particularly elevated lipoprotein(a) [Lp(a)] levels, has emerged as an important independent risk factor for ISR. In a retrospective study based on the Mayo Clinic database (*n* = 1,209; median follow-up, 8.8 years), Mahmoud et al. (17) found that the incidence of ISR was significantly higher in patients with elevated Lp(a) levels (defined as ≥50 mg/dL) than in those with lower levels (17.0% vs. 11.6%, *P* = 0.010). Elevated Lp(a) was also independently associated with long-term ISR (HR 1.67; 95% CI, 1.18–2.37; *P* = 0.004), with a stronger association observed beyond the first year after PCI. In a Chinese study that consecutively enrolled 2,086 patients undergoing PCI for ISR, Zhang et al. (18) similarly found that a baseline Lp(a) level >30 mg/dL was associated with a significantly higher risk of MACE over a median follow-up of 36 months (adjusted HR 1.31; 95% CI, 1.08–1.58; *P* = 0.007), driven mainly by all-cause mortality. Notably, the two studies used different Lp(a) thresholds (50 and 30 mg/dL), indicating that the optimal cut-off for risk stratification in patients with ISR remains to be established prospectively.

### 4.2 Novel biomarkers

The triglyceride-glucose (TyG) index, a convenient surrogate marker of insulin resistance, has attracted interest as an exploratory cardiovascular risk marker. In a cohort study of patients with chronic coronary syndrome (*n* = 1,414; median follow-up, 60 months), Guo et al. (19) found that the TyG index was associated with a composite endpoint of repeat revascularization and ISR after PCI (adjusted HR 1.191; 95% CI, 1.038–1.367; *P* = 0.013; highest versus lowest tertile, HR 1.319; 95% CI, 1.063–1.637). However, the composite combined angiographic or clinically recognized restenosis with repeat procedures, and the observational association does not establish incremental value beyond established clinical variables or demonstrate that TyG-guided management improves outcomes. The TyG index should therefore be regarded as hypothesis-generating rather than a validated tool for routine or primary-care ISR risk stratification.

The uric acid-to-albumin ratio (UAR) is another exploratory composite marker reflecting inflammation and oxidative stress. Among 503 patients treated with DES (73 with angiographic ISR and 430 without ISR), Liu et al. (20) reported an area under the receiver operating characteristic curve of 0.767 (95% CI, 0.709–0.826), with diabetes, stent length, albumin, and C-reactive protein also associated with ISR. This discrimination estimate was derived from the same study population and does not show improved calibration, reclassification, or clinical decision-making beyond established predictors. External validation and prospective assessment of clinical utility are required before UAR can be recommended for routine risk stratification.

Fibroblast activation protein (FAP)-targeted molecular imaging represents another emerging approach to the assessment of ISR. Shi et al. (21) conducted a prospective single-center imaging study in 76 symptomatic patients with coronary artery disease after PCI (30 with acute coronary syndrome and 46 with stable coronary syndrome) and 22 controls without cardiovascular disease, and found that FAPI uptake was significantly higher in ISR lesions than in non-restenotic segments compared with in-stent non-restenotic lesions (SUVmax 4.06 ± 1.91 vs. 1.69 ± 0.72, *P* < 0.0001). However, in this exploratory analysis, the association between higher FAPI uptake and MACE showed only a nonsignificant trend, and the difference in survival did not reach statistical significance. Therefore, the prognostic value of FAP-targeted imaging as a marker of ISR activity requires validation in larger prospective cohorts.

### 4.3 Lesion- and procedure-related risk factors

Anatomical and procedural risk factors for ISR include small vessel size, long lesions, calcification, bifurcations, and chronic total occlusion, as well as stent underexpansion, fracture, and geographic miss; however, the vessel diameter and lesion length thresholds used across studies are not entirely consistent. Such complex anatomical features can hinder adequate stent expansion and apposition and increase the likelihood of mechanical stent failure. Using IVUS, Yamamoto et al. (22) systematically analyzed the mechanisms of ISR in 139 ostial right coronary artery (RCA) lesions and found that biological and mechanical causes were nearly equally represented. Biological causes accounted for 53% of cases (neointimal hyperplasia, 25%; neoatherosclerosis, 22%; uncovered ostium, 6%), whereas mechanical causes accounted for 47% (stent fracture or deformation, 25%; stent underexpansion, 11%; protruding calcified nodule, 11%). The same study showed that, when mechanically mediated ISR was treated without implantation of a new stent, the subsequent event rate was 41.4%, significantly higher than the 7.8% observed under other circumstances (HR 6.44; 95% CI, 2.33–17.78). Owing to their distinctive anatomy, ostial lesions are particularly prone to stent deformation and fracture, underscoring the importance of mechanism-based interpretation under intravascular imaging guidance and adequate post-dilatation. The cumulative effects of multiple stent layers also warrant attention. A history of previous stenting is associated with a higher risk of ISR and adverse events, providing an important rationale for considering a “leave nothing behind” strategy when reintervening on ISR. In a 298-lesion IVUS study, restenotic first- and second-generation DES had smaller stent areas, longer treated segments, and more frequent stent fracture than BMS-ISR, directly supporting the contribution of underexpansion, lesion length, and structural failure (23).

## 5 Advances in diagnosis and imaging

### 5.1 Coronary angiography and intravascular imaging

Coronary angiography (CA) remains the reference standard for the anatomical diagnosis of ISR, conventionally defined as a luminal diameter stenosis of ≥50% within the stented segment or within 5 mm of its proximal or distal edges. This angiographic threshold should not be equated with an automatic indication for repeat intervention. Revascularization should be guided by symptoms, objective evidence of ischemia, lesion severity, and the clinical context. For angiographically intermediate ISR, physiological assessment with fractional flow reserve or a non-hyperemic pressure ratio may help determine functional significance when technically feasible. CA also provides only a two-dimensional representation of the vessel lumen and cannot reliably characterize the underlying biological or mechanical mechanism.

Optical coherence tomography (OCT) and intravascular ultrasound (IVUS) are currently the principal intravascular imaging modalities for mechanistic assessment of ISR, and their respective strengths are complementary. With an axial resolution of approximately 10–20 μm, OCT is particularly well suited to identifying lipid-rich neoatherosclerosis, thin fibrous caps, intraluminal thrombus, and strut coverage, whereas IVUS is better suited to assessing stent expansion, vessel dimensions, and deeper calcification. The 2023 SCAI expert consensus (4) emphasizes the use of intravascular imaging in the assessment and treatment planning of ISR to identify the underlying mechanism and optimize therapy, particularly in lesions with an unclear mechanism and in recurrent or complex ISR (Table 1,). More recently, the prospective multicenter RESTO registry showed that OCT changed the planned treatment strategy in 30% of cases; residual underexpansion after reintervention was associated with a higher 1-year target-vessel-failure rate, reinforcing the need to image both before and after treatment (24). Based on these imaging findings, a mechanism-guided algorithm for the evaluation and treatment of coronary ISR is presented in Figure 1.

**Table 1:** Intravascular imaging-based mechanistic classification of ISR and corresponding treatment strategies.

Mechanistic category on intravascular imaging	Principal imaging features	Recommended treatment strategy
Stent underexpansion	IVUS/OCT shows inadequate stent expansion; neointimal tissue or calcification may coexist	Correct the mechanical constraint with high-pressure non-compliant balloon dilatation; consider cutting/scoring balloon, IVL, or selected rescue techniques according to calcium pattern and device deliverability
Homogeneous neointimal hyperplasia	OCT shows homogeneous, high-signal, low-attenuation tissue	DCB or repeat DES after adequate preparation; comparative evidence in DES-ISR broadly favors implantation of a new stent, weighed against the cumulative number of metal layers
Neoatherosclerosis (non-calcified)	OCT shows a lipid pool, thin fibrous cap and macrophage infiltration	DCB or DES after adequate preparation; intensified lipid lowering
Calcified neoatherosclerosis	OCT/IVUS shows in-stent calcification or calcified nodules	DCB/DES after modification with IVL or a cutting/scoring balloon
Stent fracture or deformation	IVUS/OCT shows interrupted strut continuity and geometric deformation	Implantation of a new stent is usually required; In an ostial right coronary artery cohort, mechanically mediated ISR treated without implantation of a new stent was associated with a higher subsequent event rate; treatment should be guided by the underlying structural defect
Uncovered ostium	IVUS shows absence of struts in the ostial segment	Correct deformation and restore adequate lesion coverage; repeat DES is often required when scaffolding is necessary. Evidence against DCB alone is context-specific and should not be generalized from a single ostial cohort

Compiled from references (2, 4, 22–24).

**Figure 1:**
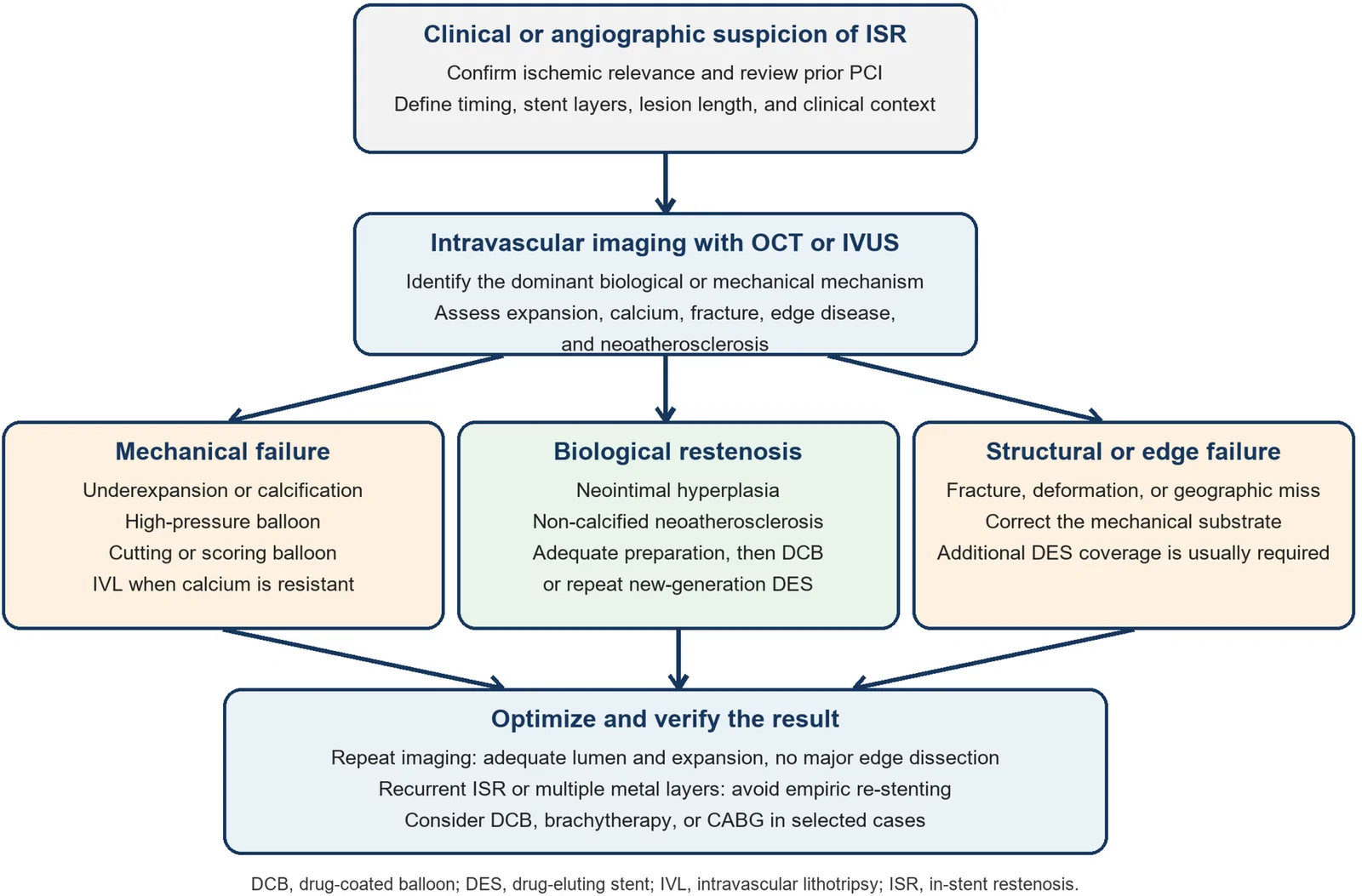
Mechanism-guided evaluation and treatment algorithm for coronary ISR.

### 5.2 Ultrahigh-resolution photon-counting CT and artificial intelligence reconstruction

Conventional coronary CT angiography (CCTA) remains limited in the assessment of ISR because of blooming artifacts generated by stent struts and is generally more suitable for the follow-up of vessels with larger-diameter stents. Ultrahigh-resolution photon-counting detector CT (UHR PCD-CT) can substantially reduce metal-related artifacts while improving spatial resolution. In a two-center retrospective study using invasive coronary angiography as the reference standard, Szilveszter et al. (25) evaluated the diagnostic performance of UHR PCD-CT for ISR in 50 patients with a total of 106 stents by comparing 0.2-mm ultrahigh-resolution reconstruction with 0.4-mm reconstruction. The proportion of evaluable stents increased from 91.5% to 99.1% with 0.2-mm reconstruction. For obstructive ISR (stenosis ≥50%), specificity (98.7% vs. 82.3%), positive predictive value (96.3% vs. 64.1%), and accuracy (98.1% vs. 84.9%) were all higher with 0.2-mm reconstruction. For non-obstructive ISR, sensitivity, specificity, and accuracy were 90.5% vs. 82.9%, 100% vs. 78.5%, and 96.2% vs. 80.2%, respectively. This advantage was also observed in the subgroup of small stents <3 mm in diameter, in which accuracy for obstructive ISR was 94.1% versus 79.4%, suggesting potential utility for the non-invasive follow-up of small-diameter stents. Deep learning-based reconstruction of CCTA has also been shown to improve the feasibility of ISR detection, suggesting that advances in image-reconstruction algorithms and CT hardware may be complementary. As PCD-CT becomes more widely available, it may offer a promising non-invasive approach for ISR assessment in selected high-risk patients after PCI. However, the available evidence is based largely on small retrospective studies, and prospective multicenter validation is required before broader clinical implementation.

## 6 Recent advances in the treatment of ISR

### 6.1 Current evidence for drug-coated balloons

By rapidly delivering an antiproliferative drug (paclitaxel or a limus analogue) to the local vessel wall, drug-coated balloons (DCBs) embody the “leave nothing behind” concept of coronary intervention by avoiding permanent metallic implants while preserving antiproliferative efficacy and potentially reducing late device-related complications, such as stent thrombosis and vasomotor dysfunction (26). Among contemporary treatment options for ISR, DCBs have accumulated one of the most extensive bodies of clinical evidence to date.

#### 6.1.1 Historical evidence and contemporary positioning

The evidence supporting DCB therapy evolved from proof-of-concept to direct comparative effectiveness. The PACCOCATH randomized trial first showed that a paclitaxel-coated balloon reduced 6-month late lumen loss and recurrent restenosis compared with an uncoated balloon in BMS-ISR (27). PEPCAD II ISR then compared a paclitaxel-coated balloon with a paclitaxel-eluting stent in BMS-ISR and demonstrated durable efficacy without another metal layer (28). In DES-ISR, ISAR-DESIRE 3 established the superiority of both paclitaxel-coated balloon angioplasty and paclitaxel-eluting stenting over plain balloon angioplasty (29), whereas RIBS IV showed superior angiographic and clinical outcomes with an everolimus-eluting stent compared with a drug-eluting balloon (30). The DAEDALUS individual-patient-data meta-analysis of 10 randomized trials (1,976 patients) found that repeat DES modestly reduced 3-year TLR compared with paclitaxel-coated balloon angioplasty, with similar composite safety outcomes; the efficacy difference was more evident in DES-ISR, whereas outcomes were more comparable in BMS-ISR (31). Thus, the type of restenosed stent matters: avoiding another metal layer is attractive in multilayer or BMS-ISR, but in single-layer ISR it may involve a modest efficacy trade-off compared with contemporary repeat DES.

#### 6.1.2 The AGENT IDE trial: landmark evidence for the paclitaxel-coated balloon

The AGENT IDE trial (32) is among the most influential recent randomized controlled trials evaluating paclitaxel-coated balloon (PCB) therapy for coronary ISR. Across 40 centers in the United States, 600 patients with ISR (lesion length <26 mm; reference vessel diameter >2.0 to ≤4.0 mm) were randomized in a 2:1 ratio to PCB (*n* = 406) or plain old balloon angioplasty (POBA; *n* = 194). The primary endpoint was 1-year target lesion failure (TLF), defined as a composite of ischemia-driven target lesion revascularization, target vessel-related myocardial infarction, and cardiac death. TLF was significantly lower with PCB than with POBA (17.9% vs. 28.6%; HR 0.59; 95% CI, 0.42–0.84; *P* = 0.003). Target lesion revascularization (13.0% vs. 24.7%; HR 0.50; *P* = 0.001) and target vessel-related myocardial infarction (5.8% vs. 11.1%; HR 0.51; *P* = 0.02) were also significantly reduced, whereas the rate of cardiac death did not differ significantly between groups (2.9% vs. 1.6%; *P* = 0.38). These findings provided pivotal clinical evidence supporting US FDA approval of the device for the treatment of coronary ISR in 2024.

A 2-year subgroup analysis of patients with and without diabetes in the AGENT IDE trial (33) showed that the difference in TLF between PCB and POBA did not reach statistical significance in either subgroup (diabetes: 29.3% vs. 35.9%; HR 0.78; 95% CI, 0.51–1.19; no diabetes: 24.2% vs. 32.2%; HR 0.66; 95% CI, 0.42–1.04). The *P* value for interaction was 0.62, indicating no statistically significant evidence that diabetes status modified the treatment effect of PCB. Point estimates favored PCB in both subgroups, although the authors explicitly noted that the analysis was underpowered. Patients with diabetes also experienced higher rates of target vessel-related myocardial infarction and TLF overall, while no stent thrombosis was observed in the PCB group.

#### 6.1.3 Accumulating evidence and reappraisal of limus-coated balloons

Limus-coated balloons (LCBs), delivering agents such as sirolimus or biolimus A9, have attracted increasing interest because they share the mechanism of mTOR pathway inhibition with new-generation DES and may offer potential advantages with respect to vascular healing. BIO ASCEND ISR (34) was a prospective, multicenter, randomized non-inferiority trial that directly compared a biolimus A9-coated balloon (BCB) with a PCB for DES-ISR. A total of 280 patients were randomized at 17 centers, with 140 assigned to each group. The primary endpoint was in-segment late lumen loss (LLL) at 9-month angiographic follow-up. LLL was 0.23 ± 0.37 mm in the BCB group and 0.25 ± 0.35 mm in the PCB group, yielding a between-group difference of −0.02 mm (95% CI, −0.12 to 0.07; *P* < 0.0001 for non-inferiority). Clinical outcomes at 12 months were comparable between the two groups, and in the OCT substudy, neointimal area at 9 months also did not differ significantly (2.32 ± 1.04 vs. 2.37 ± 0.93 mm^2^).

The 2026 SELUTION4ISR trial further informs this trade-off (35). After successful lesion predilatation, patients were randomized to a sirolimus-eluting balloon or a prerandomization usual-care strategy comprising approximately 80% repeat DES and up to 20% balloon angioplasty. The sirolimus-eluting balloon met the prespecified criterion for non-inferiority to the mixed usual-care control for 1-year target lesion failure. However, it did not establish non-inferiority to repeat DES in the single-layer ISR subgroup, and a significant interaction was observed according to whether the planned comparator was DES or balloon angioplasty. The trial therefore supports sirolimus-balloon efficacy against a mixed clinical strategy but does not justify treating limus-coated balloons as equivalent to contemporary DES for single-layer ISR.

#### 6.1.4 Systematic comparison of PCB and LCB

Recent meta-analyses of limus- versus paclitaxel-coated balloons should be interpreted according to their distinct evidence sets. Suruagy-Motta et al. (36) restricted the analysis to six randomized ISR trials (1,038 patients) and pooled different limus drugs and delivery platforms; it found a higher risk of clinically driven TLR with limus-coated balloons (RR 1.48; 95% CI, 1.02–2.14), while other clinical and angiographic endpoints were not significantly different. Sajjad et al. (37) included randomized and observational studies and combined *de novo* disease with ISR, producing a larger but clinically more heterogeneous dataset; no statistically significant difference was observed in TLF or TLR, although minimal lumen diameter was slightly smaller with sirolimus-coated balloons. These results are not directly contradictory: differences in lesion indication, study design, comparator platforms, and follow-up can dilute or modify treatment effects. Sirolimus- and biolimus-coated balloons, and their excipients or microreservoir and nanocarrier systems, should not be assumed interchangeable. Moreover, failure to detect differences in infrequent death, myocardial infarction, or thrombosis events does not establish clinical equivalence. Platform-specific randomized trials with lesion-specific analyses and adequate power for clinical endpoints remain necessary.

The prospective single-arm GINGER study provides platform-specific context for a sirolimus balloon using phospholipid nanocarriers in long *de novo* coronary lesions (38). Its angiographic and clinical findings are relevant to drug-delivery feasibility, but the absence of a comparator and the exclusive enrollment of *de novo* lesions prevent extrapolation to comparative efficacy in ISR.

### 6.2 Novel therapeutic techniques

Sequential use of paclitaxel- and sirolimus-coated balloons has been described as a “double-DCB” strategy for recurrent ISR (39). The report included only three patients, lacked a control group, and had heterogeneous follow-up; it should therefore be considered experimental and hypothesis-generating. It does not establish additive pharmacological benefit or comparative effectiveness over a single DCB, repeat DES, intracoronary brachytherapy, or surgery.

Intravascular lithotripsy (IVL) may be considered in selected ISR lesions characterized by severe calcification or resistant stent underexpansion. Acoustic pressure waves generated by IVL create fractures in calcified tissue and improve lesion compliance, thereby facilitating subsequent balloon dilatation and treatment with DCB or DES (4). Current evidence supporting IVL in ISR is derived mainly from mechanistic studies, case series, and observational data, while dedicated randomized evidence remains limited. Its use should therefore be guided by intravascular imaging to confirm a calcific mechanism and should form part of an adequately planned lesion-preparation strategy.

For selected balloon-resistant or severely underexpanded in-stent lesions, excimer laser coronary angioplasty may be used as a rescue technique in experienced centers. Vizzari et al. (40) described a stepwise contrast-enhanced excimer-laser approach that included resistant in-stent lesions. Contrast injection can amplify photomechanical effects but also increases the risk of dissection, perforation, and no-reflow; the evidence is observational, lesion selection is critical, and intravascular imaging should define the mechanism before use. This strategy should not be regarded as routine therapy or as a substitute for correction of inadequate lesion preparation at the index PCI.

### 6.3 Advances in pharmacological therapy

Beyond device-based intervention, the role of systemic pharmacotherapy in the prevention and treatment of ISR has attracted increasing attention. Among emerging strategies, the potential protective effect of sodium-glucose cotransporter 2 (SGLT2) inhibitors has generated particular interest. In an observational study of 377 patients with type 2 diabetes and acute myocardial infarction undergoing PCI, 177 of whom were receiving an SGLT2 inhibitor before the procedure, Marfella et al. (41) evaluated a composite endpoint of ISR-related cardiac death, reinfarction, and heart failure. The risk of this composite endpoint was significantly lower among SGLT2 inhibitor users than among non-users (HR 0.418; 95% CI, 0.241–0.725; *P* = 0.002). The association appeared to be independent of glycemic control and remained significant in the subgroup with HbA1c < 7% (HR 0.393; 95% CI, 0.157–0.984). Among patients who did not experience an endpoint event, stent patency at 1-year follow-up was also better in the SGLT2 inhibitor group. SGLT2 inhibitors have been proposed to exert vascular protective effects by attenuating oxidative stress, improving endothelial function, and reducing vascular inflammation. These findings suggest that their potential benefit may not be fully attributable to improved glycemic control; however, whether SGLT2 inhibitors exert direct vasoprotective or antirestenotic effects remains to be established in mechanistic studies and randomized controlled trials. Given the observational design, residual confounding, including confounding by indication, cannot be excluded, and confirmation in purpose-designed randomized controlled trials is warranted.

Intensive lipid-lowering therapy remains a cornerstone of secondary prevention after PCI. In patients with atherosclerotic cardiovascular disease at very high risk, current guidelines generally emphasize achievement of a low LDL-C level together with an adequate proportional reduction; however, an LDL-C target of <1.4 mmol/L should not itself be interpreted as evidence of an ISR-specific preventive effect. Observational studies indicate that elevated Lp(a) levels are associated with long-term ISR after PCI and with adverse outcomes following reintervention for ISR (17, 18), suggesting that residual lipid-related risk may persist despite control of conventional risk factors. RNA-targeted therapies directed against Lp(a) are currently being evaluated in cardiovascular outcome trials, but direct clinical evidence that they prevent ISR is still lacking.

## 7 Prevention of first ISR and management of recurrent ISR: future directions

### 7.1 Primary prevention: beginning in the catheterization laboratory

Optimizing the index PCI is a key strategy for reducing the subsequent risk of ISR and can be summarized into several core principles: (i) intravascular imaging guidance with IVUS or OCT to ensure appropriate stent sizing relative to the reference vessel, complete lesion coverage without significant residual disease at the stent edges, and adequate stent expansion; (ii) thorough lesion preparation to facilitate optimal stent expansion—for heavily calcified lesions, modification with a non-compliant balloon, cutting or scoring balloon, or intravascular lithotripsy may be selected according to calcium burden, calcium depth, and the degree of stent expansion achieved; (iii) judicious selection of the device strategy in small-vessel disease, with careful consideration of whether an additional metallic layer should be implanted; and (iv) minimization of total stent length and stent overlap, particularly in diffuse long lesions, with single-stent coverage preferred whenever feasible.

With regard to device selection in small-vessel disease, the network meta-analysis by Deng et al. (42), which included 39 randomized trials and 14,503 patients, provides one of the most comprehensive comparisons currently available. For target lesion revascularization, sirolimus-eluting stents (SUCRA 90.1%), zotarolimus-eluting stents (83.9%), and everolimus-eluting stents (82.2%) ranked first, second, and third, respectively. For binary restenosis, sirolimus-eluting stents, zotarolimus-eluting stents, and paclitaxel-coated balloons ranked highest, whereas conventional strategies such as bare-metal stents and plain balloon angioplasty consistently ranked lowest across endpoints. Relative to bare-metal stents, sirolimus-eluting stents reduced the risk of target lesion revascularization (OR 0.25; 95% CI, 0.15–0.43). These findings suggest that contemporary limus-eluting stents provide the most consistent overall benefit in small-vessel disease, while paclitaxel-coated balloons remain an effective alternative in selected settings. Importantly, this network meta-analysis compared device strategies rather than imaging-guided approaches; therefore, no conclusions regarding the magnitude of benefit attributable to intravascular imaging guidance can be drawn from these data.

A small two-center report evaluated a Hertz-contact calcium-modification balloon (LithiX) in 16 patients with severely calcified *de novo* lesions (43). Unlike acoustic IVL, this device generates focal mechanical stress through metallic hemispheres during balloon inflation and does not require an external generator. The series supports short-term feasibility in selected *de novo* calcified lesions but does not establish prevention of ISR or efficacy in resistant in-stent lesions. It should therefore be discussed as an emerging lesion-preparation technology rather than an evidence-based ISR therapy.

### 7.2 Secondary prevention and management of recurrent ISR

Secondary prevention after ISR treatment requires correction of both lesion-level and patient-level drivers, including verification of the final mechanical result with OCT or IVUS, intensive lipid and diabetes management, smoking cessation, and adherence to antiplatelet therapy. Recurrent ISR should not trigger automatic implantation of another stent. Imaging should identify persistent underexpansion, untreated calcium, fracture, edge disease, or biological regrowth and document the number of existing metal layers. A first recurrence with a correctable mechanical substrate may require high-pressure dilatation, calcium modification, or repeat DES when scaffolding is necessary. When adequate expansion can be achieved and another metal layer is undesirable, DCB is attractive, although comparative efficacy may be lower than repeat DES in single-layer DES-ISR. With two or more metal layers, further stenting should generally be avoided when possible because additional layers hinder expansion and are associated with recurrent failure. Intracoronary brachytherapy can be considered for refractory multilayer DES-ISR in experienced centers, while CABG should be discussed for recurrent diffuse ISR involving a major vessel, unfavorable PCI anatomy, or failure of multiple percutaneous strategies. Decisions should integrate ischemic territory, distal targets, comorbidity, surgical risk, and patient preference.

### 7.3 Bioresorbable scaffolds as an investigational prevention strategy

Bioresorbable scaffolds (BRS) are relevant to ISR primarily as a future strategy to reduce the long-term burden of permanent metallic cages at the index PCI, rather than as an established treatment for current or recurrent ISR. The rationale is to provide temporary mechanical support and subsequently undergo gradual degradation. However, pooled randomized evidence for first-generation poly-L-lactide BRS, such as ABSORB, showed higher 5-year rates of both target lesion failure (15.9% vs. 13.1%; HR 1.25; 95% CI, 1.08–1.43) and device thrombosis (2.2% vs. 1.0%; HR 2.38; 95% CI, 1.49–3.79) than everolimus-eluting metallic stents (44). Therefore, BRS should not currently be presented as a recommended ISR-prevention or treatment strategy.

Development of next-generation BRS has therefore focused on two main areas: (i) optimization of scaffold structure and manufacturing, with the central challenge of reducing strut thickness to below 100 μm while maintaining sufficient radial strength, low recoil, and fatigue resistance; and (ii) alignment of degradation kinetics with vascular healing and long-term mechanical requirements. Resorbability should not be regarded as an unconditional advantage, as the inflammatory response to degradation products, local pH changes caused by acidic by-products, the potential embolic risk associated with particle release, and the progressive loss of radial strength during resorption must all be considered (45).

## 8 Conclusion

Despite major advances in contemporary drug-eluting stent technology, coronary in-stent restenosis (ISR) remains an important mode of PCI failure and continues to pose substantial clinical challenges, particularly in recurrent and anatomically complex lesions. Current evidence increasingly supports viewing ISR as a heterogeneous disease process rather than a uniform angiographic entity. Neointimal hyperplasia, neoatherosclerosis, stent underexpansion, fracture, and other mechanical abnormalities may coexist and require distinct therapeutic approaches. Accordingly, intravascular imaging with IVUS or OCT has an important role in identifying the dominant mechanism, optimizing lesion preparation, and guiding subsequent treatment.

Drug-coated balloons and repeat drug-eluting stent implantation remain the principal evidence-based treatment options, whereas adjunctive technologies such as intravascular lithotripsy may provide additional benefit in selected lesions characterized by severe calcification or resistant stent underexpansion. Emerging molecular, metabolic, immune-inflammatory, and imaging biomarkers are providing further insights into the biological heterogeneity of ISR, although their clinical utility remains largely investigational.

Future research should move beyond device-to-device comparisons toward phenotype- and mechanism-based treatment strategies. Standardized ISR classification, imaging criteria, and clinically meaningful endpoints will be essential for the design of adequately powered randomized trials. Ultimately, integrating intravascular imaging, lesion characteristics, and patient-level biological information may facilitate a transition from empiric repeat intervention toward mechanism-guided and individualized management of coronary ISR.

## References

[1] Madhavan MV Kirtane AJ Redfors B Généreux P Ben-Yehuda O Palmerini T. Stent-related adverse events >1 year after percutaneous coronary intervention. J Am Coll Cardiol. (2020) 75(6):590–604. 10.1016/j.jacc.2019.11.058 32057373

[2] Munteanu AE Badea AA Popescu AM Pleșa FC Stanciu SM. In-stent restenosis pathophysiology and risk factors: a comprehensive review. Medicina (Kaunas). (2026) 62(2):345. 10.3390/medicina62020345 41752744 PMC12943537

[3] Elbadawi A Dang AT Mahana I Elzeneini M Alonso F Banerjee S. Outcomes of percutaneous coronary intervention for in-stent restenosis versus *de novo* lesions: a meta-analysis. J Am Heart Assoc. (2023) 12(13):e029300. 10.1161/JAHA.122.029300 37382147 PMC10356080

[4] Klein LW Nathan S Maehara A Messenger J Mintz GS Ali ZA. SCAI expert consensus statement on management of in-stent restenosis and stent thrombosis. J Soc Cardiovasc Angiogr Interv. (2023) 2(4):100971. 10.1016/j.jscai.2023.100971 39131655 PMC11308135

[5] Munteanu AE Badea AA Stanciu SM Popescu AM Pleșa FC Constantin C. Determinants of In-Sent Restenosis in ST-Elevation Myocardial Infarction: Insights from a Single-Center Retrospective Analysis. Medicina (Kaunas). (2026) 62(4):785. 10.3390/medicina62040785 42075656 PMC13117846

[6] Chen H Yuan Z Zhang S Hu X Bao J Wang G. The persisting challenge of recurrent in-stent restenosis: a systematic review and meta-analysis of prevalence and prognostic risk factors. BMC Cardiovasc Disord. (2026) 26(1):650. 10.1186/s12872-026-06016-z 42215856 PMC13422034

[7] Yi L Chen T Zhou Y Zhu P Zhu Q Shao Y. Neointimal hyperplasia and vascular restenosis: from molecular mechanisms to therapeutic interventions. Mol Biomed. (2026) 7(1):76. 10.1186/s43556-026-00477-6 42178457 PMC13199572

[8] Moses JW Leon MB Popma JJ Fitzgerald PJ Holmes DR O'Shaughnessy C. Sirolimus-eluting stents versus standard stents in patients with stenosis in a native coronary artery. N Engl J Med. (2003) 349(14):1315–23. 10.1056/NEJMoa035071 14523139

[9] Stone GW Ellis SG Cox DA Hermiller J O'Shaughnessy C Mann JT. A polymer-based, paclitaxel-eluting stent in patients with coronary artery disease. N Engl J Med. (2004) 350(3):221–31. 10.1056/NEJMoa032441 14724301

[10] An W Reyes C Espinosa-Diez C Patel P Guimaraes D Villareal F. Nitrite increases mitofusin-1 levels to inhibit vascular smooth muscle cell proliferation and prevent intimal hyperplasia. Redox Biol. (2026) 93:104169. 10.1016/j.redox.2026.104169 42033851 PMC13112402

[11] Johnson MT Trebak M. The nonexcitable smooth muscle: remodeling the smooth muscle ion transport toolkit in disease. Physiol Rev. (2026) 106(3):1757–835. 10.1152/physrev.00031.2025 41902538 PMC13218283

[12] Yang D Ni S Liu S Wang C Yang K Bi L. B lymphocytes and hyperglycemia synergistically exacerbate coronary in-stent restenosis. Int J Cardiol Cardiovasc Risk Prev. (2026) 29:200475. 10.1016/j.ijcrp.2025.200475 41815380 PMC12972726

[13] Watanabe D Marushima A Nagasaki Y Ishikawa E Matsumaru Y. Nanoparticle-based antioxidants suppress angioplasty-induced neointimal hyperplasia in rat carotid arteries. J Clin Biochem Nutr. (2026) 78(3):205–13. 10.3164/jcbn.25-231 42182940 PMC13193797

[14] Meng W Sha Y Zhong-ga C-R Pan H Zhai X Zhen P. SMPD1-mediated sphingolipid metabolism dysregulation in predicting and preventing in-stent restenosis of coronary heart disease patients. J Pharm Anal. (2026) 16(5):101553. 10.1016/j.jpha.2026.101553 42094927 PMC13141952

[15] Yan Q Fang Z Liu H. Unbiased metabolomics screening for potential biomarkers in patients with in-stent restenosis post percutaneous coronary intervention. Scand Cardiovasc J. (2026) 60(1):2613539. 10.1080/14017431.2026.2613539 41504866

[16] Nakazawa G Otsuka F Nakano M Vorpahl M Yazdani SK Ladich E. The pathology of neoatherosclerosis in human coronary implants bare-metal and drug-eluting stents. J Am Coll Cardiol. (2011) 57(11):1314–22. 10.1016/j.jacc.2011.01.011 21376502 PMC3093310

[17] Mahmoud AK Farina JM Awad K Ali NB Pereyra M Scalia IG. Lipoprotein(a) and long-term in-stent restenosis after percutaneous coronary intervention. Eur J Prev Cardiol. (2024) 31(15):1878–87. 10.1093/eurjpc/zwae212 38916491

[18] Zhang H Zhang Y Tian T Wang T Chen J Yuan J. Association between lipoprotein(a) and long-term outcomes after percutaneous coronary intervention for lesions with in-stent restenosis. J Clin Lipidol. (2023) 17(4):458–65. 10.1016/j.jacl.2023.05.094 37248114

[19] Guo X Shen R Yan S Su Y Ma L. Triglyceride-glucose index for predicting repeat revascularization and in-stent restenosis in patients with chronic coronary syndrome undergoing percutaneous coronary intervention. Cardiovasc Diabetol. (2023) 22(1):43. 10.1186/s12933-023-01779-7 36864455 PMC9983161

[20] Liu W Ding K Bao J Hu Y Gui Y Ye L. Relationship between uric acid to albumin ratio and in-stent restenosis in patients with coronary artery disease undergoing drug-eluting stenting. Coron Artery Dis. (2023) 34(8):589–94. 10.1097/MCA.0000000000001300 37855441 PMC10602219

[21] Shi X Wang L Zhang H Wu M Ren C Xue S. FAPI PET/CT in post-PCI coronary disease: in-stent restenosis characterization, MACE association, and limited short-term modulation by lipid-lowering therapy: fAPI athero trial. Eur J Nucl Med Mol Imaging. (2026) 53(10):6050–62. 10.1007/s00259-026-07970-8 42234008

[22] Yamamoto K Sato T Salem H Matsumura M Fall KN Prasad M. Mechanisms and treatment outcomes of ostial right coronary artery in-stent restenosis. EuroIntervention. (2023) 19(5):e383–93. 10.4244/EIJ-D-23-00107 37283548 PMC10397676

[23] Goto K Zhao Z Matsumura M Dohi T Kobayashi N Kirtane AJ. Mechanisms and patterns of intravascular ultrasound in-stent restenosis among bare metal stents and first- and second-generation drug-eluting stents. Am J Cardiol. (2015) 116(9):1351–7. 10.1016/j.amjcard.2015.07.058 26341188

[24] Souteyrand G Mouyen T Honton B Mulliez A Lattuca B Dilinger J-G. Stent underexpansion is an underestimated cause of intrastent restenosis: insights from RESTO registry. J Am Heart Assoc. (2024) 13(21):e036065. 10.1161/JAHA.124.036065 39450717 PMC11935687

[25] Szilveszter B Hagar MT Kulyassa P Sipos B Kubovje A Vattay B. Assessing coronary in-stent restenosis using ultrahigh-resolution photon-counting detector CT: results from a two-center study. Eur J Radiol. (2026) 199:112766. 10.1016/j.ejrad.2026.112766 41849865

[26] Giacoppo D Saucedo J Scheller B. Coronary drug-coated balloons for *de novo* and in-stent restenosis indications. J Soc Cardiovasc Angiogr Interv. (2023) 2(3):100625. 10.1016/j.jscai.2023.100625 39130710 PMC11308150

[27] Scheller B Hehrlein C Bocksch W Rutsch W Haghi D Dietz U. Treatment of coronary in-stent restenosis with a paclitaxel-coated balloon catheter. N Engl J Med. (2006) 355(20):2113–24. 10.1056/NEJMoa061254 17101615

[28] Unverdorben M Vallbracht C Cremers B Heuer H Hengstenberg C Maikowski C. Paclitaxel-coated balloon catheter versus paclitaxel-coated stent for the treatment of coronary in-stent restenosis: the three-year results of the PEPCAD II ISR study. EuroIntervention. (2015) 11(8):926–34. 10.4244/EIJY14M08_12 25169589

[29] Byrne RA Neumann F-J Mehilli J Pinieck S Wolff B Tiroch K. Paclitaxel-eluting balloons, paclitaxel-eluting stents, and balloon angioplasty in patients with restenosis after implantation of a drug-eluting stent (ISAR-DESIRE 3): a randomised, open-label trial. Lancet. (2013) 381(9865):461–7. 10.1016/S0140-6736(12)61964-3 23206837

[30] Alfonso F Pérez-Vizcayno MJ Cárdenas A García del Blanco B García-Touchard A López-Minguéz JR. A prospective randomized trial of drug-eluting balloons versus everolimus-eluting stents in patients with in-stent restenosis of drug-eluting stents: the RIBS IV randomized clinical trial. J Am Coll Cardiol. (2015) 66(1):23–33. 10.1016/j.jacc.2015.04.063 26139054

[31] Giacoppo D Alfonso F Xu B Claessen BEPM Adriaenssens T Jensen C. Paclitaxel-coated balloon angioplasty vs. Drug-eluting stenting for the treatment of coronary in-stent restenosis: a comprehensive, collaborative, individual patient data meta-analysis of 10 randomized clinical trials (DAEDALUS study). Eur Heart J. (2020) 41(38):3715–28. 10.1093/eurheartj/ehz594 31511862 PMC7706792

[32] Yeh RW Shlofmitz R Moses J Bachinsky W Dohad S Rudick S. Paclitaxel-coated balloon vs uncoated balloon for coronary in-stent restenosis: the AGENT IDE randomized clinical trial. JAMA. (2024) 331(12):1015–24. 10.1001/jama.2024.1361 38460161 PMC10924708

[33] Gaba P Jefferson BK Shlofmitz R Moses JW Bachinsky W Dohad S. Efficacy and safety of paclitaxel-coated balloon angioplasty in patients with in-stent restenosis with vs without diabetes. J Soc Cardiovasc Angiogr Interv. (2026) 5(5):105320. 10.1016/j.jscai.2026.105320 42183123 PMC13198102

[34] Chen Y Gao L Qin Q Zhang J Jia S Wu M. Biolimus-coated versus paclitaxel-coated balloons for coronary in-stent restenosis (BIO ASCEND ISR): a randomised, non-inferiority trial. EuroIntervention. (2024) 20(13):e806–17. 10.4244/EIJ-D-24-00295 38742581 PMC11200665

[35] Cutlip DE Mehran R Sharma S Doros G Jefferson B Stefanini G. Sirolimus-eluting balloon vs repeat drug-eluting stent or balloon angioplasty for coronary in-stent restenosis. J Am Coll Cardiol. (2026) 88(1):62–72. 10.1016/j.jacc.2026.03.021 41984017

[36] Suruagy-Motta RFO Carvalho PEP Da Silva LD De Oliveira Oldra LG Pereira MSS Silva LV. Limus- versus paclitaxel-coated balloons for in-stent restenosis treatment: a systematic review and study-level meta-analysis of randomized controlled trials. Am J Cardiol. (2026) 269:29–37. 10.1016/j.amjcard.2026.03.049 41887557

[37] Sajjad F Khattak AI Farhan K Arshad A Afridi A Ali MA. Sirolimus-coated versus paclitaxel-coated balloons for coronary artery disease: a grade assessed systematic review and meta-analysis. Crit Pathw Cardiol. (2026) 25(2):e0415. 10.1097/HPC.0000000000000415 41533835 PMC13193178

[38] Ielasi A Gitto M Galli S Sangiorgi GM Caminiti R Leone PP. Performance of a sirolimus-eluting balloon with phospholipid nanocarriers in the treatment of long *de novo* coronary artery disease: the prospective GINGER trial. Clin Res Cardiol. (2026). 10.1007/s00392-026-02997-1 42550208

[39] Ang AS Khaing T Khialani B Loh KKJ Ho HH. Recurrent in-stent restenosis treated with a novel technique: the double-DCB technique. JACC Case Rep. (2026) 31(21):107953. 10.1016/j.jaccas.2026.107953 42029381 PMC13221847

[40] Vizzari G Caminiti R Ielasi A Vetta G Parlavecchio A Mazzone P. Contrast-enhanced excimer laser stepwise approach during PCI for resistant coronary lesions. Catheter Cardiovasc Interv. (2024) 104(2):220–6. 10.1002/ccd.31141 38959377

[41] Marfella R Sardu C D'Onofrio N Fumagalli C Scisciola L Sasso FC. SGLT-2 inhibitors and in-stent restenosis-related events after acute myocardial infarction: an observational study in patients with type 2 diabetes. BMC Med. (2023) 21(1):71. 10.1186/s12916-023-02781-2 36829203 PMC9960194

[42] Deng C Fan C Du Z Xu Y Wu T Pan Y. Comparative effectiveness of percutaneous coronary intervention strategies for coronary small-vessel disease: a network meta-analysis of randomized trials. Ann Med. (2026) 58(1):2623329. 10.1080/07853890.2026.2623329 41631490 PMC12872083

[43] Caminiti R Ielasi A Vizzari G Marrone A Licordari R Monciino G. Real-world experience with a novel intravascular hertz contact lithotripsy balloon in severely calcified coronary lesions. JACC Adv. (2026) 5(8):102958. 10.1016/j.jacadv.2026.102958 42398440 PMC13355457

[44] Power DA Camaj A Kereiakes DJ Ellis SG Gao R Kimura T. Early and late outcomes with the absorb bioresorbable vascular scaffold: final report from the ABSORB clinical trial program. JACC Cardiovasc Interv. (2025) 18(1):1–11. 10.1016/j.jcin.2024.08.050 39814482

[45] Chen A-J Liu W Yang P Sun H Feng H Zhao HQ. Recent advances in 3D-printed bioresorbable vascular stents: toward patient-specific, novel-structured, functional, and smart devices. ACS Omega. (2026) 11(17):24941–65. 10.1021/acsomega.6c00937 42110735 PMC13150609

